# Validation of an iPad activity to measure preschool children’s food and physical activity knowledge and preferences

**DOI:** 10.1186/s12966-017-0469-z

**Published:** 2017-02-01

**Authors:** Nicola Wiseman, Neil Harris, Martin Downes

**Affiliations:** 1 Public Health, Menzies Health Institute Queensland, School of Medicine, Griffith University, Room 3.32 Building GO1, Queensland, 4222 Australia; 2 Centre for Applied Health Economics, Menzies Health Institute Queensland, School of Medicine, Griffith University, University Drive, Meadowbrook, Qld 4131 Australia

**Keywords:** Preschool children, Questionnaire, Validity, Reliability, Food, Physical activity, Knowledge, Preference, Pre-FPQ

## Abstract

**Background:**

Preschool children’s knowledge of, and preference for food and physical activity play an important role in the development of lifestyle behaviors throughout childhood. Valid and reliable instruments that are interactive and appealing to preschool children are needed, to obtain quality information in a way that actively engages children and encourages willing participation. The purpose of the current research is to assess the reliability and validity of an adapted computerized (iPad) version of the photo-pair food and exercise questionnaire (PPFEQ).

**Methods:**

The adaptation of the PPFEQ involved generating the questionnaire as an iPad-based tool, updating the photo-pairs within the questionnaire and testing for validity and reliability. This involved four phases of investigation to assess test-retest reliability, internal consistency, sensitivity to change and percent agreement of the questionnaire.

**Results:**

The adaption of the PPFEQ resulted in an 18-item questionnaire, titled the preschool food and play questionnaire (Pre-FPQ). The Pre-FPQ demonstrated acceptable reliability and sensitivity to change. Test-retest reliability and internal consistency improved with age, however, it was evident that the tool was not suitable for children younger than 4 years of age.

**Conclusions:**

Children encounter a dynamic world that shapes their knowledge, preferences, choices and behaviors. The Pre-FPQ is an innovative tool to measure preschool children’s knowledge of and preference for food and physical activity. The questionnaire offers the advantage of being presented in a well-received modality for preschool children as well as being easy and inexpensive to administer. This new tool is likely to be useful for the assessment of the effectiveness of healthy lifestyle programs implemented in the childcare setting. Future work is needed to refine and improve measures of physical activity preference in preschool children.

**Electronic supplementary material:**

The online version of this article (doi:10.1186/s12966-017-0469-z) contains supplementary material, which is available to authorized users.

## Background

Diet and physical activity are important factors in the promotion and maintenance of good health throughout the entire life course. Their role as determinants of illness and chronic disease is well established and they therefore occupy a prominent position in health promotion programs [1]. Early childhood has been identified as a critical life-stage to implement lifestyle programs, as children are beginning to establish skills and behaviors related to eating and physical activity that are often continued into adolescence and adulthood [2–4]. From a socio-ecological perspective, the development of food and physical activity behaviors by preschool children is shaped by the interaction of individual, social and environmental factors [3, 5, 6]. At the individual level, both preschool children’s knowledge of, and preference for food and physical activity play important roles in the development of lifestyle behaviors throughout childhood [3]. Accordingly, there has been growing interest in the measurement of preschool children’s knowledge of and preference for food and physical activity as possible mediators of behavior [7, 8].

### Food and activity knowledge

As children begin to gain independence, they operationalize the knowledge they have gained about food and exercise into what they perceive to be appropriate health behaviors [3]. In this way, a child’s knowledge and understanding of the benefits of a healthy diet and physical activity can have an important influence on a child’s lifestyle behaviors [9–12]. To date, very few studies have developed or applied data collection tools to specifically investigate preschool children’s knowledge of ‘healthful’ and ‘unhealthful’ physical activity [13–15]. One European study conducted by Cammisa and colleagues [15] used a play-based drawing activity to elicit the views of preschool children on physical activity and perceived barriers to practicing it; however, this tool was not tested for validity or reliability. Although several innovative and age-appropriate techniques have been used and developed to measure preschool children’s understanding of food and nutrition; few instruments have been tested for psychometric properties. Picture-based identification tasks and sorting activities (in which children sort food into groups based on type and nutritional quality) are most commonly used as an engaging and efficient technique to assess food knowledge in this age group [16, 17].

### Food and activity preferences

Food and activity preferences play an integral role in shaping lifestyle behaviors in young children. It has been suggested that children’s preferences for particular foods influence consumption more than parental intake or parental attitudes to child feeding [18, 19]. Thus, food preferences are arguably the most important predictor of young children’s food selection and consumption [7, 20–22]. There are a number of ways to measure children’s food preferences, the most effective method being the tasting method developed by Birch [7], which involves presenting children with different food products and having participants taste the items [8]. However, this method is not practical for use in large studies [21], is resource intensive, expensive and has limitations being that children can only taste a limited amount of foods before getting satiated [8]. An alternate technique which has been shown to yield similar results to tasting methods is the use of food photographs [8, 21]. Several studies have applied this method and found that good quality food photographs can effectively measure children’s food preferences as this method focuses on visual appearance, and appearance is often the first sensation to arouse interest in a given food, particularly in the case of young children [8, 21, 23–25].

In regards to physical activity preference, participation in activities that are enjoyable is also essential for a physically active lifestyle [17]. Little is known about the development of activity preferences in young children [22]. As activity preferences are related to behavior and have the potential to be changed, research to improve our understanding of the development of such preferences in children is warranted [22]. One pilot study conducted by Leary and colleagues [17], used a picture-based selection activity to test the relationship between activity preferences of parents and young children, however psychometric testing of this tool was not reported. Very few studies have examined children’s food and activity preferences simultaneously, and very rarely in preschool children [24, 26–28].

Only one tool was found to simultaneously measure preschool children’s knowledge and preference of both food and physical activity [24]. The photo pair food and activity questionnaire (PPFEQ) was designed by Calfas and colleagues in 1991 as a practical means to assess preschool children’s knowledge of and preference for physical activity and food [24]. The PPFEQ was previously validated as a paper-based picture sorting activity and has been used effectively in several studies since its development [14, 29, 30]. However, given the timeframe since the development of the PPFEQ, and the limitations identified in the initial validation of this tool, it is appropriate to update the questionnaire and determine the reliability and validity of the adapted tool.

It is increasingly recognized that instruments used to collect information from young children need to be interactive and appealing to the participant, to ensure quality information in a way that actively engages children and encourages willing participation. Computer assisted evaluation techniques are now acknowledged as being a more acceptable format for today’s children [31]. The emergence of iPad and computer assisted evaluation techniques is attractive in terms of efficiency, economy and respondent preference. The increasing use of iPads and exposure of children to iPads within the home and childcare environments make it an appealing modality for children [31]. Further, research suggests that computer-based respondents are more enthusiastic, this is important as data collected is dependent upon the respondents’ willingness to expend the effort needed to provide accurate answers [21]. The use of this format might increase motivation to proceed more carefully through the necessary cognitive processes to give an accurate answer [21, 31]. Given the advantages associated with computer-based modality of test administration, the PPFEQ was generated as an iPad application. The purpose of the current research is to assess the reliability and validity of an adapted computerized (iPad) version of the PPFEQ.

## Methods

### Procedure

The current research involved four phases of investigation. Interviews were conducted at the participating child care centers and took approximately 10 min at each time point with each child. The design of this study was guided by that used to evaluate the paper-based PPFEQ [24], as well as additional research evaluating the comparative reliability of questionnaires administered in both paper-based and computer/iPad modalities [31–33]. The adapted iPad based questionnaire is titled the *Preschool Food and Play Questionnaire* (Pre-FPQ). This study protocol was approved by the Griffith University HREC (MED/32/15/HREC).

### Phases of Investigation

#### Phase 1: Adaptation of Pre-FPQ

Alterations to the photo-pairs in the Pre-FPQ were made based on the findings of a study conducted prior to the current study, which included the use of the questionnaire [34]. Three researchers reviewed the altered/additional items (photo-pairs) to determine face validity. The questionnaire was then trialled with 10 participants to determine the appropriateness of photographs included in the tool and to determine any issues with familiarity and difficulty.

### Sample

Participants were recruited using convenience sampling at daycare centers located in Gold Coast, Australia. Parental consent was obtained for all children who participated in the study. To be eligible for inclusion in the study participants had to be aged between three and 6 years at the beginning of the study. The sample size was determined using two sample-size calculations to assess both the validity and reliability of the Pre-FPQ.

### Measures

Child food and activity knowledge and preferences were assessed with the adapted Pre-FPQ. The questionnaire consisted of 18 photo-pairs (10 food pairs and 8 activity pairs). Within these pairs, one picture represented a healthy food or activity, and the other represented a comparatively less healthy food or sedentary activity. Examples of the presentation of items in the Pre-FPQ are shown in Additional file 1. The Pre-FPQ included four subscales; food knowledge, food preference, activity knowledge and activity preference. The food scales comprised 10 photo-pairs of photographed foods representing food items from similar food categories e.g. snack items, drinks and lunch/breakfast meals. All foods were photographed on the same white plate/bowl, in some cases the food packaging was included to clarify the food type. Activity pairs were presented with the same child (aged 6) in both photos, whether male or female.

At the beginning of the test, each child was asked to choose a small doll/bear and invited to pretend they are taking care of the doll and need to help the doll to stay healthy. The doll was used so that the child would assume a caretaking role and thus make it less likely that they make choices based on personal preferences [24]. The child was then presented with 18 pairs of photographed foods or activities in random order on the iPad. The child was asked to ‘point to the food/activity that will make the doll healthy’. In order to determine preferences, the participant was then shown the 18 photo-pairs a second time and was asked to ‘point to the food or game that they like best’. The order of knowledge and preference testing was randomly assigned as a function of the iPad tool. The iPad also generated prompts at designated intervals throughout the test for the interviewer to remind participant whether they should select the food they preferred or considered the healthy alternative, this ensured consistency across interviews. Scale scores for each subscale were determined by the sum of healthful choices made (healthful choice =1 point, unhealthful/sedentary choice = 0 points).

### Phase 2: Test Reliability

During phase two of the investigation, participants were invited to complete the Pre-FPQ iPad application through one-on-one interviews with the researcher. The participants were retested after 7 days using the same protocol to determine the test-retest reliability of the Pre-FPQ.

### Phase 3: Preference validity testing

**Fig. 1:**
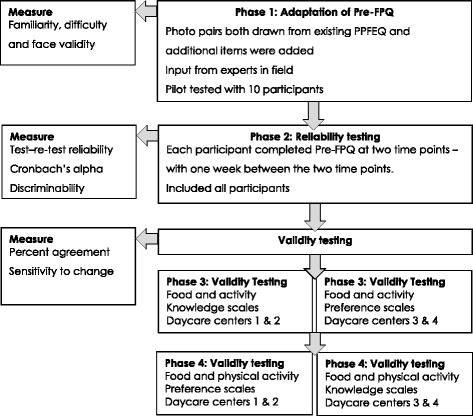
Flow diagram of study procedure

Phase three of the investigation involved the validation of the food and physical activity preference component of the questionnaire, and was conducted one week after phase one. Validity of the preference tests was determined by giving each child actual choices of the same food and activity pairs that were used in the photographs. Food pairs were presented to each child in the same way they were in the Pre-FPQ. Children were asked to select the food that they preferred. Similarly, for physical activity pairs, children were told that they could play a game for two minutes, and that they can choose between one or the other game of the activity pair. Actual food/activity preferences were compared to their stated preferences selected in the iPad questionnaire. Pairs were randomly selected for use in the preference validity tests, in an effort to reduce time burden on the participants. Half of the participants received the preference validity testing first, followed by the knowledge validity testing, this ordering was reversed for the other half of participants to control for any influences the effect order may have (see Fig. 1).

### Phase 4: Knowledge Validity testing

During the final phase of investigation, the validity of the food and activity knowledge component of the questionnaire was determined by re-administering the questionnaire after a short 10-min education session. The education session involved the explanation of very basic dietary and activity principles related to health. The sessions were made available to all children at the participating child care centers upon permission of the center director and not limited to those who returned informed consent. The sessions involved age-appropriate activities using pictures and games and did not include the use of photographs presented in the questionnaire.

### Data analysis

Statistical analyses were performed using Statistical Package for the Social Sciences (SPSS version 22.0). During the first stage of data analysis, item analysis was conducted to omit items that demonstrated poor difficulty or discriminability. Test-retest reliability of the Pre-FPQ was assessed using Pearson correlation tests by comparing the average score of food knowledge, food preference, physical activity knowledge, and physical activity preference scores from stage one and stage two of the investigation. Validity of the knowledge scales of the questionnaire were assessed by calculating the extent of the improvement on knowledge scores following the brief intervention. Paired-samples t-tests were used to determine any significant changes in knowledge scores. A *p* value of <0.05 was considered statistically significant. Validity and reliability were also calculated separately for each age group (3, 4 and 5 years), due to subgroup analysis a Bonferroni correction was used and a p value less than 0.016 was considered statistically significant. The preference scales were validated by calculating the percent agreement (and its 95% confidence interval) between stated preference and actual choices of foods and activities.

## Results

A total of 18 photo pairs were included in the final Pre-FPQ. Food and activities selected for the photo-pairs were drawn from the validated paper-based version of the tool; several of the items were updated after pilot testing due to low familiarity. Given the extended timeframe between the development of the PPFEQ and the current study, photo-pairs within the tool were updated to be more contemporary. For example, due to the emergence of new technologies since the questionnaire was last tested for validity and reliability, activities involving screen-time (e.g. television viewing, computer gaming) were added to the physical activity component of the questionnaire as sedentary alternatives [22]. There were four pairs retained from the original tool, eight pairs were altered and six pairs were added to the questionnaire (see Table 1).

**Table 1:** Photo-pairs used to assess food and activity knowledge and preference

Healthful/Active	Unhealthful/Sedentary
Activities	
1. Cricket^a^	Reading
2. Skipping^a^	Watching Television
3. Soccer^a^	Checkers
4. Swimming^a^	Computer
5. Running^b^	iPad
6. Climbing^b^	Xbox
7. Bike^b^	Legos
8. T-ball^b^	Coloring
Food	
1. Sultanas	Mixed lollies
2. Rice	Chips
3. Yoghurt^a^	Doughnuts
4. Salad sandwich^a^	Jam sandwich
5. Water^a^	Juice
6. Oranges^b^	Cookies
7. Rice cakes	Crisps
8. Weetbix	Frootloops
7. Milk^a^	Cola
10. Apples^b^	Cake

^a^= Pair was added to Pre-FPQ

^b^ = Pair was altered from item in original PPFEQ

A total of 86 Australian preschool children (44 boys and 42 girls) participated in the study. The age of participants ranged from 37 months to 66 months (M = 51 months, SD 7.5). In the initial stage of the analysis, item analyses were conducted to identify any items that demonstrated poor discriminability or test-retest reliability. The Pre-FPQ can be broken down into the following four subscales: food knowledge (Score out of 10), physical activity knowledge (8), food preference (10) and physical activity preference (8). The mean and standard deviation of subscale scores are presented by age in Table 2. Participant’s knowledge of both food and physical activity increased with age, whereas food and physical activity preference remained relatively consistent across each age group.

**Table 2:** Mean (SD) of the subscale scores of the Pre-FPQ by age group

Subscale	Total *N* = 86	3 Years *N* = 27	4 Years *N* = 40	5 years *N* = 19
Food knowledge score^a^	4.56 (2.75)	3.33 (1.84)	4.67 (2.84)	6.38 (2.98)
Food preference score^a^	3.97 (2.14)	3.52 (1.76)	4.43 (2.42)	3.56 (1.86)
Activity knowledge score^b^	4.71 (1.82)	4.59 (1.76)	4.62 (1.71)	5.19 (2.28)
Activity preference score^b^	4.34 (1.59)	4.56 (1.80)	4.17 (1.62)	4.31 (1.13)

^a=^score range 1-10 ^b=^score range 1- 8

**Fig. 2:**
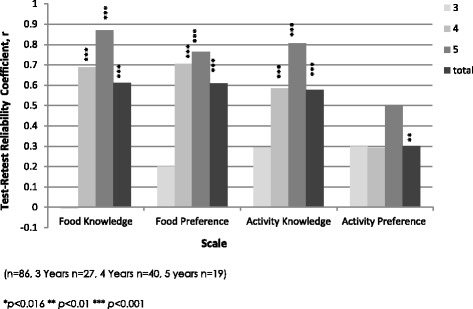
Test-retest reliability of each subscale of the Pre-FPQ for 3, 4 and 5 year old participants

**Fig. 3:**
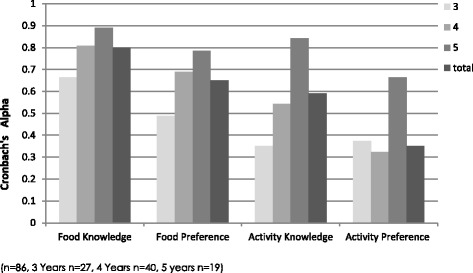
Internal consistency of each subscale scale of the Pre-FPQ for 3, 4 and 5 year old participants

Pearson correlation tests demonstrated that the test-retest reliability for food knowledge (*r* =0.61, *p* = <0.001), food preference (*r* = 0.61. *P* < 0.001) and for physical activity knowledge (*r* =0.58, *p* = <0.001) was moderate and for physical activity preference (*r* =0.30, *p* = <0.01) (Fig. 2) was poor. Developmental effects were assessed by comparing results in children aged three, four, and five. Figure 2 shows that the reliability of each knowledge scale improved with the age of participant. Food and activity knowledge scales and the food preference scale demonstrated higher reliability in comparison to the activity preference scale. Alpha coefficients for food knowledge (0.80) and food preference (0.65) demonstrated good and acceptable internal consistency respectively. Alpha coefficients were low for physical activity knowledge (0.59) and preference (0.35), though this improved with the age of participants (Fig. 2). Overall, internal consistency improved with participant age for all scales with the exception of physical activity preference (Fig. 3).

**Fig. 4:**
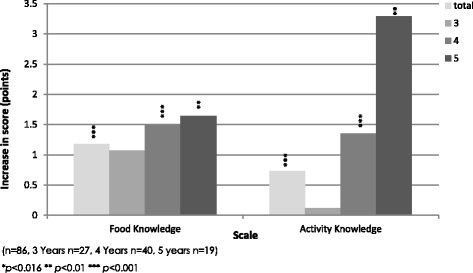
Improvement in food and physical activity knowledge scores after education session by age group

The validity of the food and activity knowledge scales were tested using paired samples t-tests to determine the sensitivity of scales to change by testing for any improvements in knowledge following a short education session. Overall, the questionnaire measured significant improvements in participant’s physical activity knowledge (*t* = -3.40, *p* = <0.001) and food knowledge (*t* =4.18, *p* = <0.001). Improvements in knowledge increased with age and demonstrated no validity in the 3-year-old children (See Fig. 4).

**Fig. 5:**
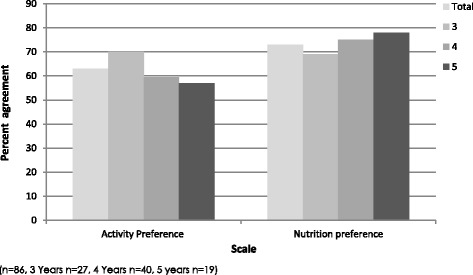
Percent agreement between stated preference and actual food and activity choice by age group

The preference scales were validated by calculating the percent agreement between stated preference and actual choices of foods and activities. The percent agreement between stated food preference and actual food choices was 73%, again this improved with age with the percent agreement being 78% in 5-year-old children. Percent agreement between stated activity and physical activity preferences was 63%; however, percent agreement declined with age (See Fig. 5).

## Discussion

On the whole, the reliability and sensitivity of the Pre-FPQ was acceptable. Test-retest reliability and internal consistency improved with age; however, it was evident that the tool was not suitable for children younger than 4 years of age. Validity of the food and activity knowledge scales was indicated by significant increases in children’s knowledge scores after exposure to a healthy lifestyle education program. The percent agreement between stated food preference and choice of food was high. In contrast, the percent agreement between activity preferences and choice of physical activity was relatively low and was inconsistent across age groups. It was evident that situational factors played a role in influencing children self-reported physical activity preferences.

Food knowledge scales demonstrated high test-retest reliability and good internal consistency, comparing well with similar tools measuring food knowledge in the same population [35–37]. The responsiveness of the questionnaire to change in knowledge was an encouraging finding, as sensitivity to change is essential if a questionnaire is to evaluate the effectiveness of an intervention [38]. The percent agreement between food preference and actual choices was high, this reinforces literature suggesting that the use of photos as a method to test children’s food preferences can be an adequate substitute to tasting methods, and offers a practical means to test large samples of participants [8, 21, 23–25]. Test-retest reliability of the food preference scales also compared well to studies that utilized the tasting technique of preference testing [8, 21, 23–25]. Test-retest reliability and internal consistency of food preference scales improved with the age of participants, demonstrating high internal consistency for 4 and 5-year-old children. This is consistent with a study conducted by Guthrie and colleagues [23], which found a clear trend in differences in reliability by age with significantly lower reliability in 3 year olds compared with 5 year olds.

Although reliability and validity for the physical activity preferences scale were low, the physical activity knowledge scale demonstrated high test-retest reliability and good internal consistency for 5 year olds. Further, the questionnaire measured significant improvements in participant’s physical activity knowledge after children were exposed to a short educational intervention, which indicated the scale is sensitive to changes in physical activity knowledge. Whilst the results of this study indicate physical activity scales are less reliable than food scales, the adapted Pre-FPQ offers a proximal and direct way to measure children physical activity preferences [39, 40]. To date, no tool is available to measure self-reported physical activity preferences in preschool children thus making the Pre-FPQ a valuable addition to existing literature. This reflects emerging literature emphasizing the importance of actively engaging young children in the research process, rather than relying solely on the recounts of their caregivers [40]. Nonetheless, more work needs to be done to refine the physical activity preference component of the tool.

Interestingly, Calfas and colleagues [24] had a similar finding in that; the percent agreement between activity preferences and choice of physical activity was relatively low in their validation of the PPFEQ. Although the adapted tool indicated improved percent agreement of the physical activity preference scale, low and inconsistent levels of percent agreement may reflect a lack of concrete physical activity preferences in this age group [17]. Alpha coefficients for physical activity preference were low, however, they demonstrated acceptable internal consistency for children that were 5 years of age. Research on preschooler physical activity preferences is limited, mostly being measured through parental reporting [17, 24, 38, 39]. Thus, it is not possible to compare the reliability and validity of this component of the Pre-FPQ with existing measures.

In an effort to explain the low reliability of the physical activity preference scale in comparison to the food preference scale, Calfas and colleagues suggested that children’s choice of activity may be more situation dependent than their choice of food [24]. For example, as children’s food preferences may remain relatively stable over time, children’s physical activity preferences may be influenced by the context in which they were tested, and what they feel like doing on that occasion. Interestingly, this was observed in the current study through comments of participating children when measuring their activity preferences. Activity choices were influenced by the weather at the time of testing, the novelty of the activity (e.g. the use of iPad) and the activity being completed in the classroom prior to testing. There is a need for future research to consider how situational factors influence a child's self-reported physical activity preferences, and to develop a means to control for such influences.

Another key finding of this study was that the Pre-FPQ does not appear to be appropriate for the measurement of lifestyle knowledge in 3-year-old participants, as no significant changes in knowledge were observed after the intervention. Although evidence suggests that 3-year-old children have the cognitive ability to understand and retain information regarding healthy food and physical activity [3], this was not reflected by the findings of this study. This may indicate that more specific techniques are needed to measure food and activity knowledge and preferences in 3-year-old children, such as less direct, observational methods [41].

The modality of the adapted Pre-FPQ offered several advantages over the paper-based version of the questionnaire. Using pictures in a computerized iPad format facilitated a fast and reliable measurement of food and physical activity preferences and knowledge in preschool children. The tool was easy to use, inexpensive and required little time and effort from participants, which helped the researcher to work within the attention span of the target age-group. The iPad modality of the Pre-FPQ was an appealing and exciting format that enabled the researcher to engage and maintain the concentration of participants. Further, the tool was able to be applied quickly to large numbers of participants and allowed for results to be exported securely and efficiently to a data analysis program to assist with timely analysis.

The study was also subject to limitations. The sample size was not large enough to examine the interactions between age and methods rigorously, although patterns of results are consistent across the study and can be seen as a good indication of the influence of age on the tool. In addition, the sample of participants for this study was drawn from childcare centers residing in middle-to-high socioeconomic neighborhoods. These limitations restrict the generalizability of the study findings.

## Conclusions

This study adds to the literature by providing a validated means to measure preschool children’s knowledge and preference for food and physical activity. The Pre-FPQ is an innovative tool and demonstrated adequate reliability and validity for the assessment of food and activity knowledge and preferences in 4-5 year-old children. It was evident that the tool was not appropriate for children younger than 4 years of age. The Pre-FPQ offers the advantage of being presented in a well-received modality for preschool children as well as easy and inexpensive to administer. This new tool is likely to be useful for the assessment of the effectiveness of healthy lifestyle programs implemented in the childcare setting, which aim to encourage the development of healthy dietary and activity behavior in young children. Future work is needed to refine and improve measures of physical activity preference in preschool children.

## Additional file

Additional file 1: Screenshot examples of Pre-FPQ. (DOCX 1458 kb)
