# Supplementary figures and images for: Validation of an iPad activity to measure preschool children’s food and physical activity knowledge and preferences
Source: Int J Behav Nutr Phys Act. 2017 Feb 1;14:11. doi: 10.1186/s12966-017-0469-z (PMC5286816; doi:10.1186/s12966-017-0469-z)

**Appendix 1. Screenshot examples of Pre-FPQ**


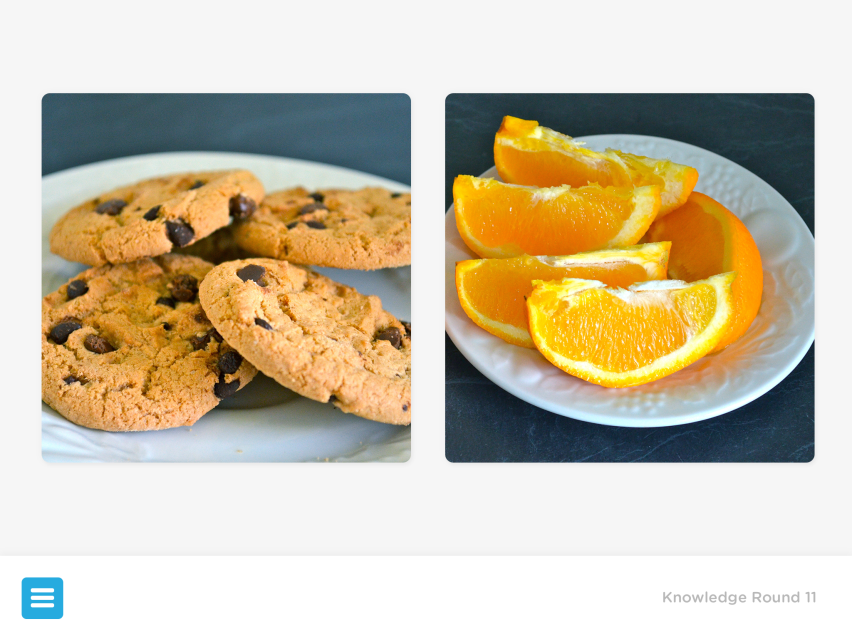


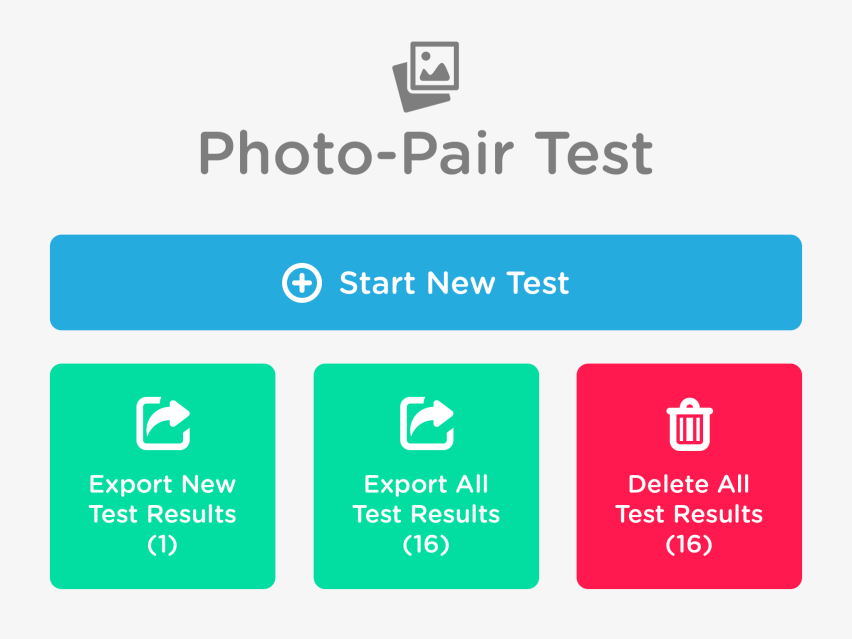


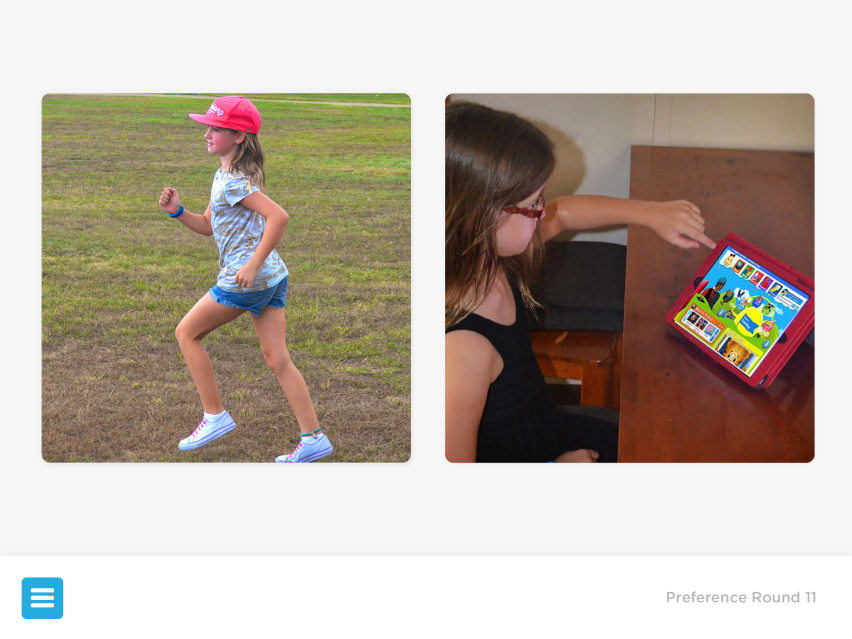

Supplement: Additional file 1: — Screenshot examples of Pre-FPQ. (DOCX 1458 kb) [file 12966_2017_469_MOESM1_ESM.docx]
